# A 20 MHz Repetition Rate, Sub-Picosecond Ti–Sapphire Laser for Fiber Delivery in Nonlinear Microscopy of the Skin

**DOI:** 10.3390/life14020231

**Published:** 2024-02-07

**Authors:** Ádám Krolopp, Luca Fésűs, Gergely Szipőcs, Norbert Wikonkál, Róbert Szipőcs

**Affiliations:** 1 HUN-REN Wigner RCP, Institute for Solid State Physics and Optics, P.O. Box 49, H-1525 Budapest, Hungary; 2 R&D Ultrafast Lasers Ltd., Konkoly-Thege Street 29-33, H-1121 Budapest, Hungary; 3 Department of Dermatology, Venereology and Dermatooncology, Semmelweis University, Mária Street 41, H-1085 Budapest, Hungary

**Keywords:** nonlinear optical microscopy, second-harmonic generation, two-photon excitation fluorescence, skin cancer, adult hemangioma

## Abstract

Nonlinear microscopy (NM) enables us to investigate the morphology or monitor the physiological processes of the skin through the use of ultrafast lasers. Fiber (or fiber-coupled) lasers are of great interest because they can easily be combined with a handheld, scanning nonlinear microscope. This latter feature greatly increases the utility of NM for pre-clinical applications and in vivo tissue imaging. Here, we present a fiber-coupled, sub-ps Ti–sapphire laser system being optimized for in vivo, stain-free, 3D imaging of skin alterations with a low thermal load of the skin. The laser is pumped by a low-cost, 2.1 W, 532 nm pump laser and delivers 0.5–1 ps, high-peak-power pulses at a ~20 MHz repetition rate. The spectral bandwidth of the laser is below 2 nm, which results in a low sensitivity for dispersion during fiber delivery. The reduction in the peak intensity due to the increased pulse duration is compensated by the lower repetition rate of our laser. In our proof-of-concept imaging experiments, a ~1.8 m long, commercial hollow-core photonic bandgap fiber was used for fiber delivery. Fresh and frozen skin biopsies of different skin alterations (e.g., adult hemangioma, basal cell cancer) and an unaffected control were used for high-quality, two-photon excitation fluorescence microscopy (2PEF) and second-harmonic generation (SHG) z-stack (3D) imaging.

## 1. Introduction

Nonlinear microscopy, such as two-photon excitation fluorescence microscopy (2PEF) [1,2], second-harmonic generation (SHG) microscopy [3,4] and coherent anti-Stokes Raman scattering (CARS) microscopy [5,6], is increasingly used to perform non-invasive, in vivo studies in life sciences. These techniques enable us to investigate the morphology [7,8,9,10,11,12] or monitor the physiological processes (e.g., monitoring drug delivery) of the skin [13,14,15] through the use of ultrafast pulse lasers. Recent years have brought revolutionary progress in the development of sub-ps pulse, all-fiber laser oscillators and amplifiers that are suitable for nonlinear microscopy. Fiber (or fiber-coupled) lasers are of great interest because they can easily be combined with endoscopy [16,17,18,19,20]. This latter feature greatly increases the utility of nonlinear microscopy for pre-clinical applications and tissue imaging. In 2016, we reported on a novel, handheld 2PEF/SHG microscope imaging system comprising a sub-ps, ~2 MHz repetition rate Yb–fiber laser system [21], which was suitable for the in vivo imaging of murine skin at an average power level of as low as 5 mW at a 200 kHz sampling rate (corresponding to a 5 µs pixel dwell time value). The whole nonlinear microscope imaging system had the main advantages of a low price for the sub-ps laser, fiber optics flexibility, a relatively small, light-weight scanning and detection head and a very low risk of thermal or photochemical damage of the skin samples [22,23].

In principle, 2PEF (or time-resolved 2PE fluorescence lifetime imaging (FLIM)) microscopy can visualize endogenous fluorophores, such as elastin, keratin, NADH, FAD, etc., while the morphology of collagen fibers can be assessed by SHG microscopy. Due to the 1030 nm operation wavelength of our Yb–fiber laser system, however, we could not efficiently excite a few of these endogenous fluorophores (such as elastic fibers, NADH and FAD) with our handheld 2PEF imaging system. This fact considerably limited its applicability in cases of rare skin diseases (such as Ehlers–Danlos syndrome (EDS) [24] and pseudoxanthoma elasticum (PXE) [25,26]) or in cases of basal cell carcinoma (BCC) [27,28,29]. The latter is not only the most common skin tumor, but it is also the most common malignancy in Caucasians. Note that in cases of a table-top nonlinear microscope system, a commercial, broadly tunable, ~100 fs Ti–sapphire system [30], whose operation wavelength can be easily set in the 680–1040 nm range and where each of the above-mentioned fluorophores can be efficiently 2P-excited, is typically used for 2PEF or SHG imaging.

During the last couple of years, there has been further progress in (i) reducing the size and price of ultrashort pulse laser oscillators used for TPEF, SHG and FLIM imaging at around 800 nm [31,32] and (ii) increasing the imaging area offered by multiphoton imaging [21,26,31,32]. In Refs. [31,32], a frequency-doubled Er–fiber laser (Carmel X-series, Calmar Laser, Palo Alto, CA, USA) was used for imaging; the laser could be mounted directly to the scan head due to its reduced size of 9 × 18 × 3.5 cm^3^. The laser delivers 90 fs pulses at 780 nm at an 80 MHz repetition rate with a free-space laser output (no fiber delivery option), since the 780 nm light is generated by a bulk optics SHG unit at the Er-fiber amplifier fiber end. We must note, however, that due to its size, weight and relatively high spectral bandwidth, such a frequency-doubled Er–fiber laser still cannot be integrated into our handheld 2PEF/SHG microscope imaging system [21]. In general, the imaging area of a scanning nonlinear microscope system with sub-micron spatial resolution is limited to around 0.5 × 0.5 mm^2^, which must be extended to the cm^2^ range for clinical applications. The combination of optical and mechanical scanning mechanisms reported in Refs. [21,26,31,32] offers a possible solution to this problem. In Refs. [31,32], a small-size piezo stage was applied for “mosaic” imaging of different skin samples in a cm^2^ area, while in Refs. [21,26], a step-motor-driven x–y stage was used. In the former case, the imaging contrast between different endogenous fluorophores was efficiently increased by time-resolved single-photon counting (SPC) [31,32]. To the same end, the spectral decomposition of the three-channel detection of 2PEF data was applied in Ref. [26].

In order to overcome the problems related to the fixed 1030 nm operation wavelength of our Yb–fiber laser used for 2PEF and SHG imaging in our experiment reported in Ref. [21], we decided to replace it with a fiber-coupled Ti–sapphire laser that can be operated in the 790–820 nm range, whose physical parameters (repetition rate, spectral bandwidth, peak intensity, etc.) were optimized for fiber delivery, low thermal loads and in vivo imaging of the skin. Similar systems reported earlier [33,34,35] applied hollow-core photonic bandgap fibers in combination with a sub-100 fs, 76 MHz repetition rate Ti–sapphire laser and were sensitive to the dispersive elements applied (e.g., Pockels cells, microscope objective and beam-steering mirrors [36]), the operation wavelength of the laser [33] and the length and birefringence of the hollow-core fiber [37]. Most of these problems might be solved by the application of dispersion-compensating [38,39] reversed-dispersion-slope hollow-core photonic bandgap fibers [40], but they are not commercially available and might have a similar problem of birefringence, resulting in double optical pulses at the fiber end in case of sub-100 fs pulses [33]. In this paper, we report on a fiber-coupled, low-repetition-rate, sub-ps Ti–sapphire laser system that is free from all of these problems and allows for fiber delivery with minimum temporal [33] and spatial [40,41] distortion of the optical pulses. The system was used to examine healthy skin, adult hemangioma and basal cell cancer in ex vivo skin biopsies.

## 2. Laser Setup and Fiber Delivery Experiments

The ~20 MHz repetition rate (long-cavity), sub-ps Ti–sapphire laser used in our fiber delivery and nonlinear microscope imaging experiment was similar to that that we had used for real-time histology of the skin by simultaneous CARS imaging of lipids and proteins [42]. Its long-cavity configuration laser comprising a Herriott cell is described in detail in Ref. [43]. For our fiber delivery (and CARS) imaging experiments, however, we had to make a few modifications to the cavity dispersion and self-amplitude modulation in order to reduce the spectral bandwidth of the laser and to improve the mode-locking performance, respectively. A lower spectral bandwidth results in a higher chemical selectivity in DVRF-CARS imaging [42] and a lower sensitivity of optical pulses to dispersive effects during fiber delivery. To this end, we replaced the intra-cavity SF10 prism pair compressor with a piezo-controlled Gires–Tournois interferometer (GTI) [44], which provided considerably higher intra-cavity dispersion than the prism pair previously used. Due to its critical role, the design, construction and performance of this special-dispersion-compensating device will be discussed in detail later.

**Figure 1:**
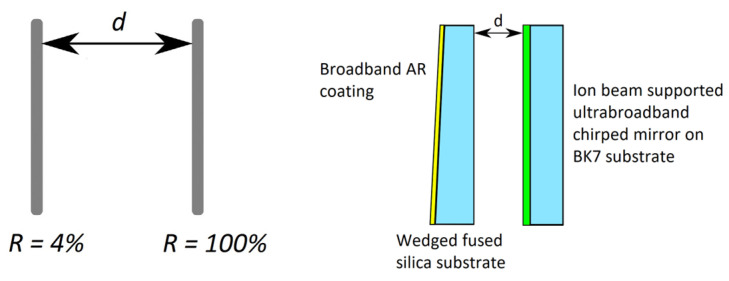
Theoretical model (**left**) and physical layout (**right**) of our piezo-controlled GTI that can be used for dispersion compensation all over the high reflectivity range of UBCM mirror (680–1040 nm). Light blue: glass substrates, green: ultrabroadband chirped mirror, yellow: broadband antireflection coating.

The theoretical model and physical layout of the GTI are shown in Figure 1 (left and right). For our experiments, a support fine mechanics for an ion-beam-sputtered (IBS) ultrabroadband chirped mirror (UBCM) [30] with a thickness of 6.35 mm, diameter of 12.7 mm (product of R&D Ultrafast Lasers Ltd., Budapest, Hungary) and a rectangular, slightly wedged (wedge angle: 1.04 degree) fused silica substrate was designed. Separation between the highly reflective surface of the UBCM and the uncoated surface of the fused silica wedge was controlled by 3 piezo actuators (Polaris P20, product of Thorlabs Inc., Newton, NJ, USA). The travel distance of this piezo actuator was ~15 μm for a piezo control voltage of 150 V. The outer, wedged surface of the fused silica substrate was antireflection-coated for the whole tuning range of the Ti–sapphire lasers in order to minimize intra-cavity losses. Fresnel reflection at the air–fused silica interface resulted in a ~4%, wavelength-independent reflection acting as the partial reflector of the GTI, while our ion-beam-sputtered ultrabroadband chirped mirror was a nearly ideal high reflector, providing a wavelength-independent high reflectance of R > 99.9% over the full tuning range (680–1040 nm) of the Ti–sapphire lasers [30].

**Figure 2:**
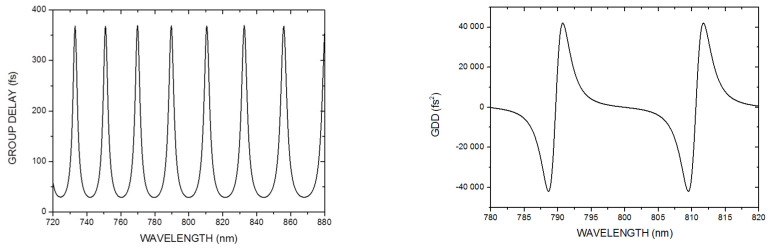
Computed group delay (**left**) and GDD (**right**) vs. wavelength functions of a GTI mirror with mirror spacing of *d* = 15 μm.

The maximum amount of dispersion provided by a GTI depends on the reflectance of the partial mirror and the mirror separation *d*, while the free spectral range between two group delay maxima is determined only by the mirror separation *d* (see Figure 1). The computed group delay and group delay dispersion (GDD) vs. wavelength functions corresponding to a GTI with a partial mirror reflectance of 4% and a physical mirror spacing of *d* = 15 µm are plotted in Figure 2.

For a mirror separation of *d* = 15 μm, we found that the free spectral range of the GTI was at around 20 nm, which required the fine setting of mirror spacing *d* in order to provide negative dispersion at a certain operational wavelength. Note that a Brewster angled birefringent filter (BRF) element was built in the laser cavity for the fine tuning of the operational wavelength of the laser.

**Figure 3:**
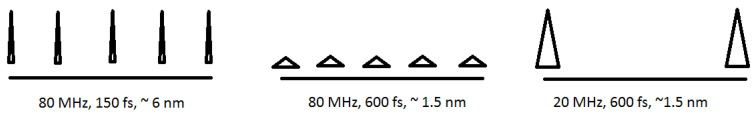
Basic idea of our laser setup for distortion-free fiber delivery of high-peak-power ultrashort pulses for nonlinear microscopy. Enlarged intra-cavity dispersion resulted in spectrally narrower, longer optical pulses with reduced peak intensity, which was compensated by lower repetition rate of our Ti–sapphire laser system, resulting in high signal-to-noise ratio in vivo imaging at an average power of as low as ~20 mW.

It could also be observed that at the short wavelength side of a resonance wavelength (which corresponds to one of the group delay maxima), the dispersion of a GTI mirror was in the range from −20,000 fs^2^ to −40,000 fs^2^, which must be compared to that of a previously used SF10 prism compressor (GDD ~ −2000 fs^2^ to −6000 fs^2^). Owing to the enlarged net intra-cavity dispersion, the spectral bandwidth of the long-cavity Ti–sapphire laser was reduced to below 2 nm at typical intra-cavity peak powers. Accordingly, the pulse duration of the laser increased above 0.5–0.6 ps. This nearly four-fold reduction in the peak intensity was compensated by the lower (~20 MHz) repetition rate of our long-cavity Ti–sapphire laser compared to industry standard (~76–80 MHz), mode-locked ultrafast Ti–sapphire lasers delivering ~100–150 fs pulses. The basic idea for the reduction in the effect of dispersion during the fiber delivery while keeping the 2-photon excitation efficiency is illustrated in Figure 3.

Our GTI-controlled, long-cavity Ti–sapphire laser is pumped by a cost-efficient, 2.1 W average power, 532 nm laser (Opus, product of Laser Quantum, Manchester, UK). Accordingly, the overall cost of this fiber-coupled, ultrashort laser operating at around 810 nm is comparable to that of a ~2 MHz Yb–fiber laser previously used for in vivo nonlinear microscopy of the skin [21]. Note that a commercial Ti–sapphire laser typically used for nonlinear microscopy comprises a from 10 to 14 W, 532 nm laser as a pump laser, which is a critical price factor in the overall cost of an NM system.

The tuning range of our current setup is currently limited by the finite reflection band (780 to 820 nm) of a single SAM-800 saturable absorber mirror (BATOP GmbH, Jena, Germany). Experiments are in progress to utilize the full tuning range offered by the Ti–sapphire gain medium and the IBS UBCM mirrors by the combination of several SAM devices optimized for different wavelength regimes of our interest.

**Figure 4:**
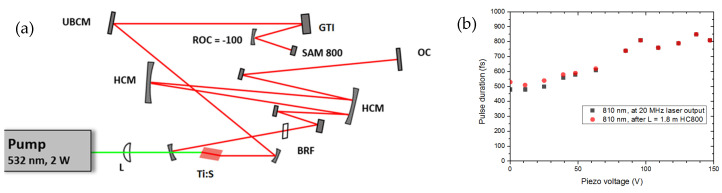
(**a**) Setup of the 20 MHz repetition rate sub-ps Ti–sapphire laser used for fiber delivery and nonlinear microscope imaging of the skin. Ti:S: Ti–sapphire crystal (path length: PL = 4 mm), BRF: birefringent filter for wavelength tuning, HCM: Herriott cell mirrors, UBCM: ultrabroadband chirped mirrors, GTI: a piezo-controlled Gires–Tournois interferometer, SAM 800: saturable absorber mirror, OC: output coupler. (**b**) Pulse duration of the laser vs. piezo voltage of GTI measured before (black dots) and after (red dots) a 1.8 m hollow-core fiber. A higher piezo voltage resulted in a higher mirror spacing (i.e., higher negative dispersion value at the GDD maximum) of the GTI at the laser wavelength. Green line: 532 nm pump beam, red line: Ti–sapphire laser beam.

The final configuration of the ~20 MHz repetition rate, sub-ps Ti–sapphire laser used for the fiber delivery and imaging experiment is shown in Figure 4a. The pulse duration of the ~20 MHz laser was characterized by a PulseCheck autocorrelator (APE GmbH, Berlin, Germany). Depending on the intra-cavity dispersion set by the mirror spacing of an intra-cavity GTI, the pulse duration could be set in the 0.5–1 ps range (see Figure 4b, black dots). Note that a higher *d* value (see Figure 1) resulted in higher GDD peak values. In principle, the pulse duration vs. piezo voltage (which should be set for the maximum GDD at around a certain peak) function should be linear, since the GDD peak value increases linearly with the mirror spacing *d*. However, in practice the pulse duration is determined by the actual GDD value and is also affected by the intra-cavity pulse energy, which depends on transmission/scattering losses of the GTI for a certain piezo setting. This explains the slight deviation of our measured data from our expected linear function (see Figure 4b, black dots).

The Δλ < 2 nm spectral bandwidth of the laser allows for the distortion-free fiber delivery of optical pulses (over the whole tuning range of the laser) through a ~1.8 m long HC-800-2-type, commercial, hollow-core photonic bandgap fiber with a honeycomb structure (product of NKT Photonics A/S, Birkerod, Denmark) (see Figure 4b, red dots). Note that a HC-800-2 fiber has its zero-dispersion wavelength at around 780 nm, according to its datasheet. The mode-locked average power of the laser is ~225 mW at an 810 nm operation wavelength. After fiber coupling and decoupling, it was reduced to ~125 mW due to coupling losses in our experiment.

## 3. Imaging Results

For our imaging experiments, we used a commercial Axio Examiner LSM 7 MP laser scanning 2P microscope (Carl Zeiss, Jena, Germany), the detection optics of which were also optimized for the background-free SHG imaging of collagen at around 405 nm. The laser beam of our 20 MHz repetition rate sub-ps Ti–sapphire laser was focused by a 20× water immersion objective (W-Plan—APOCHROMAT 20×/1.0 DIC (UV) VIS–IR, (Carl Zeiss, Jena, Germany), which resulted in an approximately 0.6 × 0.6 mm^2^ imaging area and a ~0.5 μm spatial resolution in the x–y direction and a 1.5 μm one in the z direction.

Fresh and frozen skin biopsies of healthy skin, as well as adult hemangioma and basal cell carcinoma (BCC) were used for testing our fiber-coupled laser for z-stack imaging.

**Figure 5:**
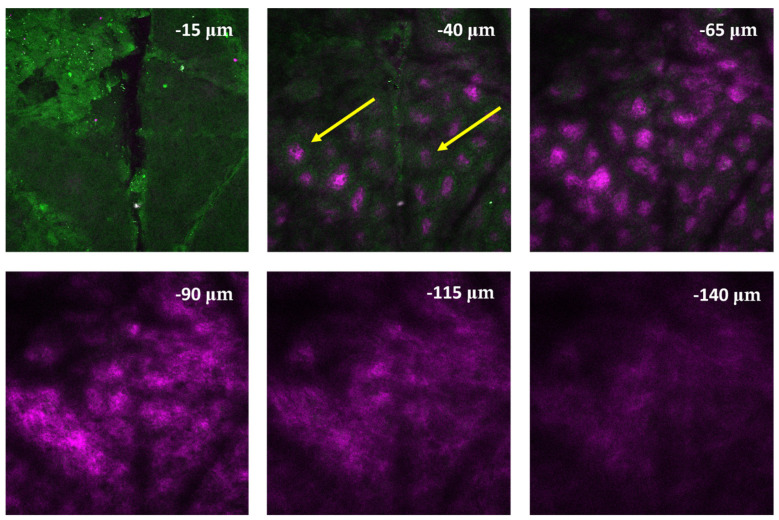
Z-stack image of a healthy skin biopsy from the bottom (lower right image) to the top (upper left image) of the biopsy. The first slice at −15 µm shows the stratum corneum. Below, the stratum basale is indicated (yellow arrow), which is located along the papillary dermis. Below, fine collagen fibers of the papillary dermis are visible (−65 µm). SHG signal of collagen was collected with a 405/20 nm emission filter (magenta) and 2PEF signal of keratin was collected using a 500–550 nm (green) emission filter. Laser central wavelength was 810 nm, and the average power on the sample was ~20 mW. Image size: 420 × 420 µm^2^. Z-stack imaging depth was measured from the surface of the skin sample.

In case of the healthy skin biopsy (see Figure 5), the keratinized layer of the stratum corneum appeared first with a homogeneous green (500–550 nm) fluorescence. Then, the individual cells of the stratum granulosum were discovered with intracellular keratin granules. Below, the stratum basale could be distinguished, which constitutes the deepest confluent layer of the epidermis above the papillary dermis. Finally, fine collagen fibers appeared in the dermal papillae (shown by its SHG signal in violet).

**Figure 6:**
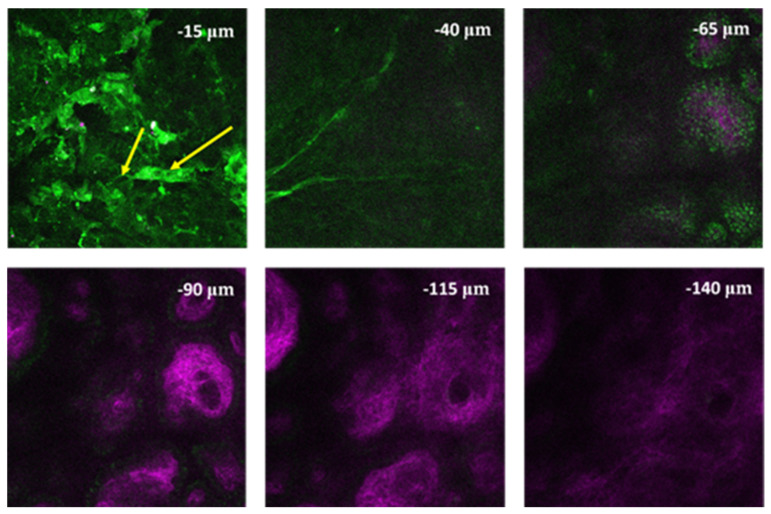
Z-stack image of an adult hemangioma skin biopsy from the bottom (lower right corner) to the top (upper left corner) of the biopsy. Yellow arrow: longitudinal and cross-sectional circular appearance of capillary proliferation. In deeper layers, SHG signal of collagen appeared. Z-stack imaging depth was measured from the surface of the skin sample. Other imaging parameters were the same as those in Figure 5.

In the case of adult hemangioma [45] (see Figure 6), we could observe an increased superficial 2PEF signal in a circular arrangement, which corresponded to capillaries. In addition, the epithelium showed a normal structure.

**Figure 7:**
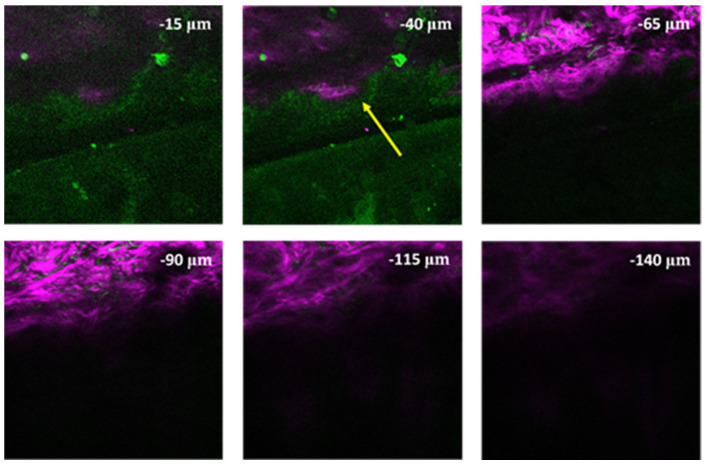
Z-stack image of a skin biopsy of a basal cell cancer from the bottom (lower right image) to the top (upper left image) of the biopsy. Yellow arrow: the green 2PEF signal that arose from keratin highlights the palisade cells at the tumor border. Inside the tumor, the homogeneous basal cell morphology appeared. In the deeper layers, the magenta SHG signal of collagen became visible around the tumor nest. Z-stack imaging depth was measured from the surface of the skin sample. Other imaging parameters were the same as those in Figure 5 and Figure 6.

In the case of BCC [28], we could detect the tumor mass by a strong contrast of 2PEF arising from cells with a basal cell morphology throughout the whole tumor (see Figure 7). In addition, the border of the tumor was highlighted by the palisade orientation of these cells. We could also observe a strong SHG signal of collagen fibers that surrounded the tumor nest.

## 4. Discussion

In this paper, we presented a fiber-coupled, sub-ps Ti–sapphire laser system that was optimized for in vivo, stain-free, 3D imaging of skin alterations with a low thermal load on skin samples. The laser was pumped by a low-cost, 2.1 W, 532 nm pump laser and delivered 0.5–1 ps, high-peak-power pulses at a ~20 MHz repetition rate with an average power of ~200 mW at around 800 nm. The laser had a low sensitivity for fiber dispersion, since its spectral bandwidth was below 2 nm. Using a 1.8 m long, commercial hollow-core fiber for delivery, the pulse duration and mode-field distribution of the laser practically did not change, which allowed us to combine it with a handheld NM imaging device [21].

We tested this fiber-coupled, low-repetition-rate Ti–sapphire laser to record NM images of different skin biopsies with imaging depths of 200 μm at an average power as low as 20 mW on the skin surface.

In the case of the healthy skin sample, most of the epithelial layers could be identified, and the connective tissue fibers in the papillary dermis were also visible.

Adult hemangioma or cherry angioma is a common benign vascular proliferation in the skin. It appears as 1–5 mm bright-red papules, mostly on the trunk and the upper limbs. It consists of narrow capillaries with enlarged endothelial cells [45]. In the case of adult hemangioma, we could observe the circular endothelial wall of these capillaries throughout the epidermis and papillary dermis. For clinicians, it is important to differentiate hemangioma from angiokeratoma, which is an acquired benign vascular malformation and a cutaneous sign of Fabry’s disease, an X-linked genodermatoses with severe systemic organ involvement and diverse clinical symptoms [46]. Basal cell cancer (BCC) is the most common malignancy among white individuals. Its estimated lifetime prevalence is 30% [47]. With nonlinear microscopy, we could detect the cells with a basal cell morphology comprising the tumor mass and the cells with peripheral palisading at the edges of the tumor. These are also the histopathological hallmarks of BCC that enable diagnosis [48].

The proposed system enabled subcellular resolution imaging, where the individual cellular morphology was clearly identifiable within the upper 100 μm. Although this imaging depth does not qualify the methodology for the assessment of the thickness of a skin tumor, it is suitable to determine the type and dignity of the lesion. For a clinician, this is of utmost importance. In the case of a malignant skin lesion, the gold-standard therapy is surgical excision. Determining the tumor border prior to surgery would lead to less incomplete excision, which can cause recurrence and can lead to complicated cosmetic and therapeutic challenges. The presented laser system combined with a handheld imaging device [21] is a cost-efficient, technically more robust alternative to a sophisticated, dual-wavelength CARS imaging system that is suitable for quasi-H&E-stained images for the detection of BCC [12,42] or any other NM system comprising any commercial, 80 MHz repetition rate, ~100 fs pulse Ti–sapphire laser system [7,12].

Finally, we must also emphasize that there exist other 3D non-invasive grayscale imaging technologies for living skin imaging, such as reflectance confocal microscopy (RCM) and optical coherence tomography (OCT), but they do not offer the chemical selectivity of nonlinear microscopy [31,32]. Additionally, we must also mention that frequency-doubled Er–fiber lasers (such as the FemtoFiber smart 780 from Toptica [49], Munich, Germany, or the Carmel X-series from Calmar Laser, Palo Alto, CA, USA [31,32]) might be alternatives to the Ti–sapphire laser system presented in this paper at a similar cost, but they still have a higher sensitivity for fiber dispersion and no tuning option that might be critical technical issues. For instance, the latter might be an important factor in in vivo NM to avoid possible thermal damage [22,23] (due to the low excitation efficiency for certain fluorophores such as NAD(P)H and FAD) or photo-chemical damage [23] (e.g., the formation of cyclobutane pyrimidine dimers (CPD) in DNA) during exposure of skin samples to lasers, and our fiber-coupled, tunable sub-ps Ti–sapphire laser offers this tuning option in contrast to frequency-doubled, fixed-wavelength Er–fiber lasers.

It is out of the scope of this paper, but we must also mention that metabolic imaging of the skin using free and bound NAD(P)H fluorescence detection by FLIM is also an important, rapidly evolving research area that can be efficiently applied for determining tumor borders [50] and to follow the effect of drug treatment on diabetes-related skin alterations such as ulcers or to investigate the depth-dependent keratinocyte metabolism [51] or mithochondrial dynamics in human skin for diagnosis [52]. Metabolic imaging, however, presents further technical issues, such as the elevated imaging time for accurate TCSPC systems or other practical problems related to SPC.

## Data Availability

The data presented in this study are available on request from the corresponding author.
